# Capacity for Study in Children

**Published:** 1881-05

**Authors:** 


### CAPACITY FOR STUDY IN CHILDREN.

Chadwick, the best authority (says the Boston *Medical and Surgical Journal*) on the above topic, concludes that a child from the age of five to seven can attend to one subject for fifteen minutes; from seven to ten years about twenty minutes; from ten to twelve, about twenty-five minutes; from twelve to eighteen, about thirty minutes. The total daily mental work suitable for a young person from twelve to sixteen years of age, taking Chadwick's estimate as a basis, and making allowance for the rapid development in this period, is placed at from five to six hours.—*The Sanitary News.*

